# Geographic Regions and Environmental Factors Influence the Nutritional and Flavor Quality of Newhall Navel Oranges

**DOI:** 10.3390/foods15213792

**Published:** 2026-10-23

**Authors:** Zhen Wang, Mengnan Tan, Ning Cao, Yixuan Fan, Xinyu Zhao, Shaoxiang Pan, Ye Song, Xinhuan Yan, Xuemei Liu, Qi Guo, Maoyu Wu, Yong Peng, Xiaodong Zheng

**Affiliations:** 1 Jinan Fruit Research Institute, All China Federation of Supply & Marketing Co-Operatives, Jinan 250014, China; wangz033026@163.com (Z.W.);; 2 College of Food Science and Engineering, Shandong Agricultural University, Tai’an 271018, China

**Keywords:** Newhall navel oranges, nutritional qualities, aroma substances, different producing regions

## Abstract

Newhall navel oranges show distinct quality differences across major producing regions in China but their specific quality characteristics remain unclear. This study compared their sensory, nutritional, and flavor properties, and investigated environmental effects and related underlying mechanisms. The results showed that Newhall navel oranges from Xinfeng, Jiangxi Province, achieved the highest overall sensory score, characterized by high sucrose content, richer aroma, and greater consumer preference. In contrast, those from Zigui, Hubei Province, exhibited higher acidity alongside greater levels of functional components (ascorbic acid, hesperidin, β-carotene). Further analysis identified (E)-2-hexenal, ethyl butanoate, octanal-D, hexanal-D, ethyl acetate, and pentan-2-one as differential aroma markers across producing regions. Hesperidin and ascorbic acid were significantly positively correlated with altitude but negatively correlated with temperature and light time, whereas sucrose content showed a negative correlatedion with altitude. Variations in the fruit quality of Newhall navel oranges were significantly correlated with climatic conditions: higher altitude and lower temperature may be associated with elevated ascorbic acid contents, while greater precipitation may correlate with reduced sugar concentrations. This study provides indirect evidence for the influence of geographical regions and environmental factors on the quality differences of Newhall navel oranges.

## 1. Introduction

The Newhall navel orange *(Citrus sinensis* (L.) Osbeck) is an early-maturing bud sport of the Washington navel orange, first selected in California, USA. Since its introduction into China in 1978, this cultivar has been widely accepted by consumers because of its early maturity, attractive appearance, round fruit shape, and superior eating quality [1]. It is currently cultivated on a large scale in Jiangxi, Hubei, and Chongqing, the main production areas in China, with additional plantings in Guangxi, Fujian, Zhejiang, Yunnan, Guizhou, Guangdong, and Taiwan; among these, the Gannan region of southern Jiangxi Province is the most renowned [2]. Newhall navel oranges are rich in organic acids, vitamins, and minerals, and contain abundant bioactive compounds such as carotenoids, flavonoids, and polyphenols, which exhibit strong antioxidant capacity and have been associated with a reduced risk of cardiovascular disease and certain cancers [3,4].

The quality of Newhall navel oranges depends on variety and producing regions [5]. Unique geographical and climatic conditions in different regions—such as sunlight, diurnal temperature variation, and soil properties—significantly influence sugar content, acidity, flavor, and other quality attributes [6,7]. Adequate sunlight promotes sugar synthesis and peel coloration, while substantial diurnal temperature variation facilitates sugar accumulation; soil factors notably affect the accumulation of mineral elements in the fruit. Furthermore, climate and soil collectively regulate the synthesis and metabolism of flavor compounds [8,9]. It is these environmentally driven variations across regions that collectively shape the final external appearance and internal flavor profile of Newhall navel oranges.

Renowned for their excellent flavor and storage quality, Newhall navel oranges are highly favored by consumers [10]. Research on the Newhall navel orange has primarily addressed breeding, cultivation practices, postharvest preservation, and processing characteristics [11,12], whereas geographical variation in fruit quality has received increasing attention in recent years. Hu compared the physical characteristics and chemical compositions (including soluble solids, titratable acidity, sugars, organic acids, minerals, amino acids, and vitamin C) of Newhall navel oranges from several major production areas in China and examined their relationships with climatic variables such as temperature, rainfall, and sunshine duration [13]. More recently, Shu [14] applied untargeted metabolomics and nutritional evaluation to geographical-indication Newhall navel oranges from Ganzhou, Fengjie, and Zigui, with eight orchards sampled per region. And a further study compared the physicochemical and volatile characteristics of Newhall navel oranges from 13 production areas in relation to meteorological factors [15].

Nevertheless, the above quality dimensions have so far been investigated individually or only in partial combination, and the production areas of Xingshan (Hubei) and Xinfeng (Jiangxi) have not been characterized within any of these frameworks. The novelty of the present study therefore lies in three aspects: (i) to our knowledge, this is the first study to integrate, within a single analytical framework and on the same sample set, descriptive sensory profiling, functional components (hesperidin, ascorbic acid, and β-carotene), a complete mineral profile including five heavy metals, and HS-GC-IMS volatile fingerprinting coupled with OPLS-DA-based marker identification for Newhall navel oranges; (ii) it provides the first multidimensional quality characterization of Newhall navel oranges from Xingshan and Pingyuan; and (iii) it explores, in an explicitly hypothesis-generating manner, the associations between this multidimensional quality dataset and the environmental gradient across production areas. Accordingly, Newhall navel oranges from five major production areas in China (Xinfeng in Jiangxi Province, Zigui and Xingshan in Hubei Province, Fengjie in Chongqing Municipality, and Pingyuan in Guangdong Province) were used as experimental materials to evaluate differences in sensory, nutritional, and flavor qualities, to identify key markers distinguishing these production areas, and to investigate the correlations between fruit quality and environmental factors. The results of this study provide a reference for geographical origin identification and consumer guidance, as well as a basis for optimizing cultivation strategies for Newhall navel oranges under climate change.

## 2. Materials and Methods

### 2.1. Plant Selection and Treatments

**Figure 1:**
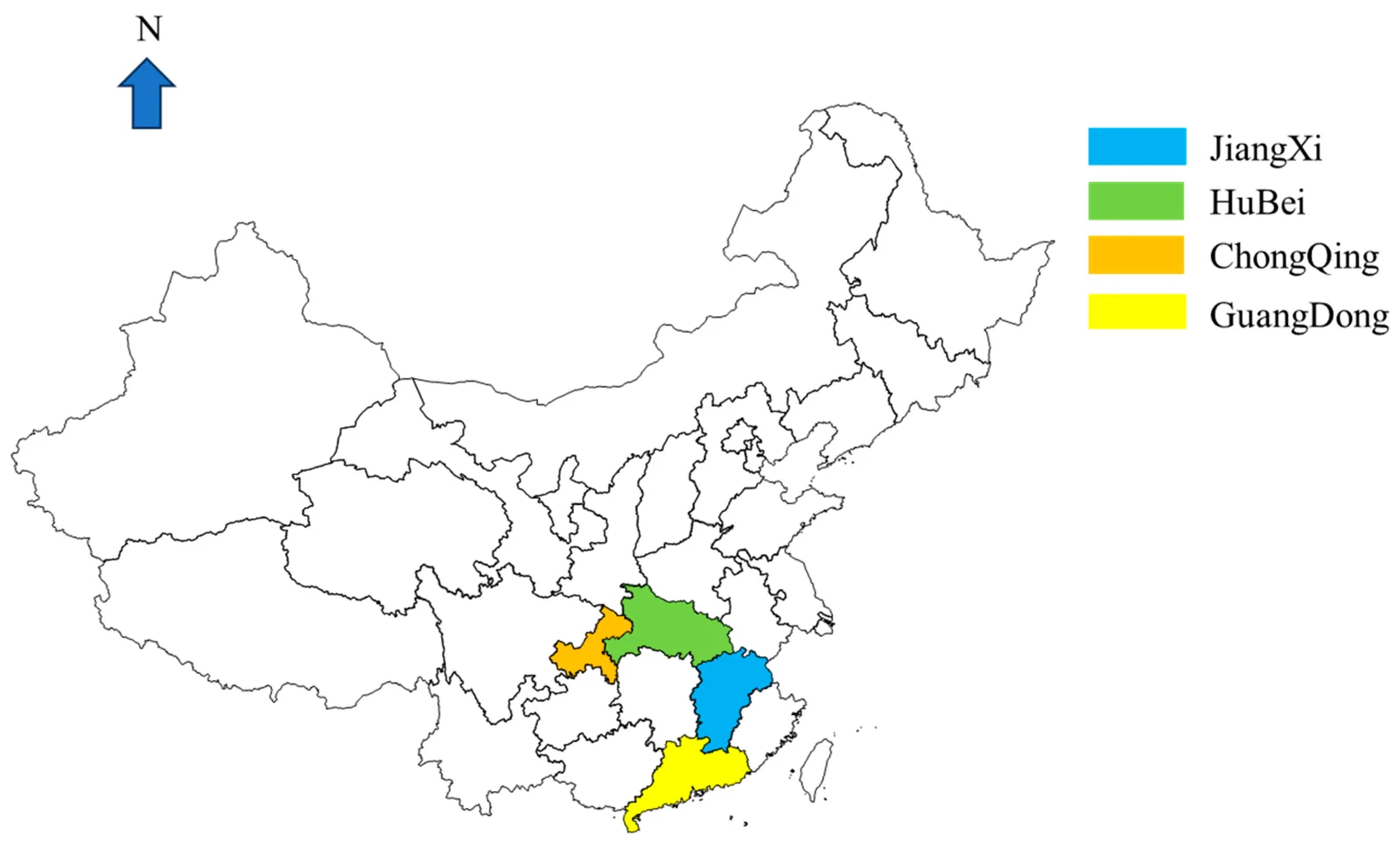
A geographical distribution map of the Newhall navel orange-producing regions.

Newhall navel orange samples were collected from five major producing regions: Xinfeng, Jiangxi Province (Xinfeng Anxi Chengzhiyuan Navel Orange Cooperative) (Ganzhou, Jiangxi, China); Zigui, Hubei Province (Zigui Qingtan Taoyecheng producing and Marketing Cooperative) (Yichang, Hubei, China); Xingshan, Hubei Province (Xingshan Zhaojun Fruit Industry Co., Ltd.) (Yichang, Hubei, China); Fengjie, Chongqing Municipality (Fengjie Kuikan Navel Orange Planting Cooperative) (Fengjie, Chongqing, China); and Pingyuan, Guangdong Province (Pingyuan Chengzhixiang Fruit Industry Cooperative) (Meizhou, Guangdong, China). One representative orchard was sampled per region, meaning the orchard represents the regional sampling unit rather than independent biological replicates across orchards. These sampled orchards are representative sites of their respective producing regions and are located within the legally defined protected zones for the corresponding geographical-indication products. Detailed geographical information of the sampling sites is provided in Figure 1 and Table 1. The selected orchards from each production region shared largely consistent management practices, including fertilization regimes, irrigation strategies, pest and disease prevention, and general orchard maintenance. All sampled trees were 13–14 years old. To minimize confounding variations, sampling was collectively performed in October 2023. Random sampling was adopted for tree and fruit selection within each orchard. For each orchard, five healthy trees with uniform vigor and cropping performance were randomly selected across different canopy orientations. Ten fruits were harvested from the upper, middle and lower canopy layers of each tree, resulting in thirty fruits per tree. In total, one hundred and fifty fruits (five trees × thirty fruits per tree) were collected from the three canopy positions to form one composite sample representative of the orchard. After harvest, all fruits were immediately transported to the laboratory under cold-chain conditions at 4 °C. Upon arrival, fruits of uniform size free from mechanical damage, pests and diseases were randomly selected from each regional batch for sensory evaluation and physicochemical analysis. For the determination of nutritional and other physicochemical indicators, eight fruits were randomly selected for individual sample preparation from each producing region. The selected fresh fruits were peeled and homogenized with a blender (Guangdong, China), and the fruit puree was analyzed immediately after preparation. The samples were labeled as follows: JXXF for Jiangxi Xinfeng, HBZG for Hubei Zigui, HBXS for Hubei Xingshan, CQFJ for Chongqing Fengjie, and GDPY for Guangdong Pingyuan.

**Figure 2:**
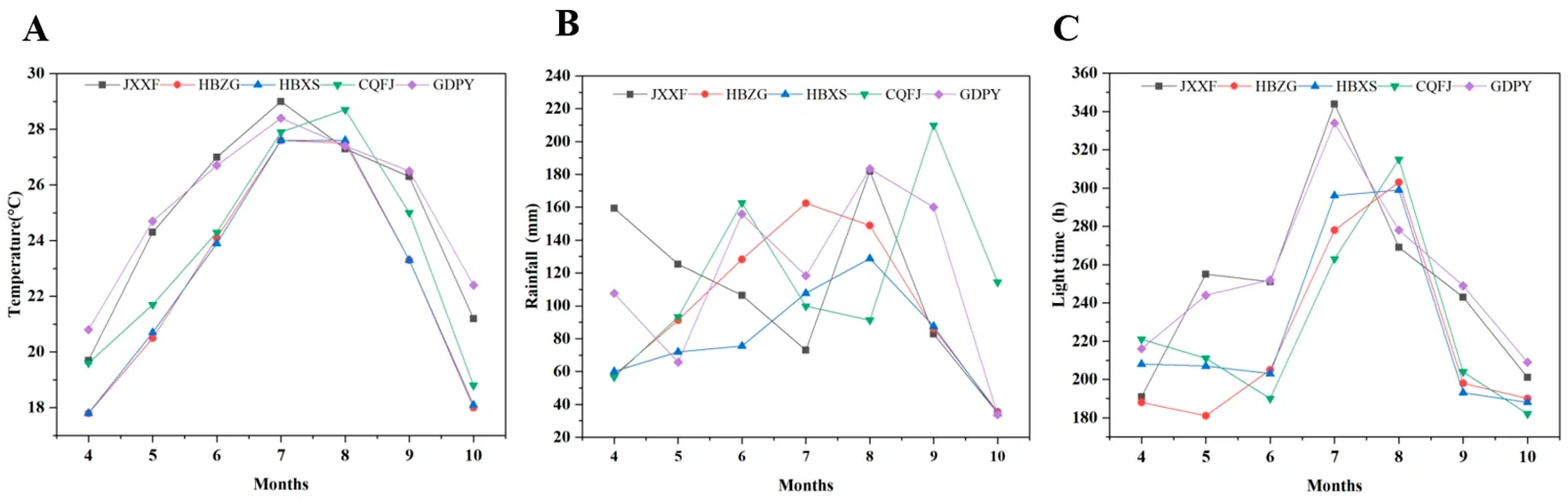
Climate information on Newhall navel oranges in different production regions. (**A**) Temperature. (**B**) Rainfall. (**C**) Light time.

Meteorological data including temperature, rainfall, and sunshine duration during the period from bud burst through flowering to fruit harvest in 2023 across the five producing regions are provided in Figure 2. These climate data were obtained from the China Meteorological Administration and local meteorological yearbooks.

### 2.2. Chemicals and Reagents

Malic acid, citric acid, fructose, glucose, sucrose, hesperidin, and β-carotene were obtained from Shanghai Yuanye Biotechnology Co., Ltd. (Shanghai, China) Methanol and acetonitrile were HPLC grade (≥99%) and obtained from TEDIA Co., Ltd. (Fairfield, OH, USA). All other analytical-grade chemical reagents were obtained from Tianjin Kaitong Chemical Reagent Co., Ltd. (Tianjin, China).

### 2.3. Sensory Evaluation

The sensory evaluation panel consisted of 15 trained members (7 males and 8 females, with an average age of 32). Panelists were recruited according to basic screening criteria including normal taste and olfactory function, no history of allergic rhinitis, and no smoking habit. The training lasted for 4 weeks, with two sessions per week. During training, panelists familiarized themselves with the sensory descriptors and reference standards of Newhall navel oranges. Consistency tests were performed at the end of training to verify panel reliability, and only panelists who passed the consistency test participated in formal evaluation. Through group discussion, we developed a consensus-based sensory evaluation sheet (Table S1 in Supplementary Materials), which included detailed description scales for each attribute intensity.

All sensory tests were carried out in a standardized sensory evaluation room maintained at 22–24 °C and 50–60% relative humidity. Fruit samples were prepared uniformly, coded with random three-digit numbers, and randomly assigned to panelists with balanced presentation order to reduce sequence bias. Samples were served at room temperature (22–24 °C). Each evaluation session was arranged with adequate intervals between samples; purified water was provided for panelists to rinse their mouths between samples to reduce carry-over effects and sensory fatigue. Three replicate evaluation sessions were performed.

For the descriptive analysis, panelists rated the perceived intensity of six descriptive attributes (sweetness, acidity, bitterness, aroma, juiciness, and pulp texture) on a 5-point intensity scale, with higher scores indicating stronger perceived intensity of each attribute. Separately, overall liking was assessed in an independent hedonic test on a 5-point liking scale, with higher scores indicating a greater degree of liking. We have clearly distinguished descriptive attribute intensity measurement from hedonic preference assessment in the text to avoid confusing these two different sensory response types. In addition, the statistical analysis methods for sensory data have been supplemented in Table S2.

Ethical approval for sensory evaluation was not necessary at our institution, the Jinan Fruit Research Institute, All China Federation of Supply & Marketing Co-Operatives. All participants provided written informed consent prior to participating in the sensory evaluation. In addition, the rights and privacy of all participants were safeguarded throughout the study.

### 2.4. Peel Thickness, Edible Rate, Juice Yield and Color Measurement

Peel thickness was determined according to the group standard T/JTGJ 001-2020 [16] http://down.foodmate.net/standard/sort/12/92295.html (accessed on 13 October 2024). Edible rate and juice yield were measured according to the Chinese national standard GB/T 8210-2011 [17] http://down.foodmate.net/standard/sort/3/28679.html (accessed on 13 October 2024). Colorimetric values of Newhall navel oranges were measured using a chroma meter (Tokyo, Japan). Measurements were taken on the equatorial region of the fruit, as well as on the juice extracted from the pulp. Each fruit was measured three times, and six fruits were evaluated per producing region. Here, L* represents lightness, with higher values indicating greater brightness; a* represents the degree of red (+) or green (--), and b* represents the degree of yellowness (+) or blueness (-). All measurements were performed in three biological replicates, and results were reported as means ± standard deviation.

### 2.5. Determination of Total Soluble Solids (TSS), Soluble Sugar (SS), Titratable Acid (TA), Organic Acids (OA) and Ratio of Total Soluble Solids to Titratable Acidity (RTT)

TSS and TA were determined in accordance with the Chinese national standard GB/T 8210-2011 http://down.foodmate.net/standard/sort/3/28679.html (accessed on 15 October 2024). SS was analyzed according to method I of the Chinese national standard GB 5009.8-2023 [18] http://down.foodmate.net/standard/sort/3/145876.html (accessed on 13 October 2024). The results are expressed as g/100 g fresh weight (FW). OA were analyzed referring to the Chinese national standard GB 5009.157-2016 [19] http://down.foodmate.net/standard/sort/3/49341.html (accessed on 15 October 2024). The results are expressed as g/kg FW. The ratio of total soluble solids to titratable acidity (RTT) is the ratio of TSS to TA. All measurements were conducted in three analytical replicates, and the results are presented as means ± standard deviation.

### 2.6. Total Hesperidin Content

Hesperidin was determined by high performance liquid chromatography (HPLC), following the Chinese agricultural standard NY/T 2014-2011 [20] http://down.foodmate.net/standard/sort/5/36654.html (accessed on 15 October 2024). Briefly, 10 g of homogenate was weighed into a volumetric flask. Ammonium oxalate solution was added, followed by dimethylformamide. The mixture was homogenized, diluted to volume with water, and transferred to a conical flask. The flask was placed in a water bath for 10 min and cooled to room temperature. The supernatant was collected and filtered through a 0.45 µm membrane filter prior to instrumental analysis. The analysis was performed on a Thermo U3000 system (Waltham, MA, USA) consisting of a UV detector, a binary gradient pump, and an Acclaim C18 column (4.6 mm × 250 mm, 5 µm). The mobile phase consisted of 0.5% acetic acid (A) and methanol (B) under isocratic elution at a flow rate of 0.8 mL/min. The column temperature was maintained at 35 °C ± 1 °C, the injection volume was 10 μL, and the detection wavelength was 283 nm. Hesperidin was identified by comparison with the retention time and UV spectra of the standard compound. The calibration curve was *y* = 0.66 + 0.35*x*, R^2^ = 0.9992, where “*y*” represents the concentration of hesperidin in mg/L, and “*x*” denotes the peak area. The calibration curve for hesperidin was established over the linear range of 1.0 mg/L to 200 mg/L. The extraction recovery was 95.8%. Under repeatability conditions, the absolute difference between two independent determination results shall not exceed 10% of their arithmetic mean. The limit of detection (LOD) was 0.3 mg/kg, and the limit of quantification (LOQ) was 1 mg/kg. The content of hesperidin was expressed as mg/kg FW. All measurements were conducted in three analytical replicates, and the results are presented as means ± standard deviation.

### 2.7. Determination of β-Carotene

β-carotene was determined according to the Chinese national food safety standard GB 5009.83-2016 [21] http://down.foodmate.net/standard/sort/3/50427.html (accessed on 15 October 2024). Briefly, 5 g of homogenate was weighed into a conical flask. After the addition of ascorbic acid and anhydrous ethanol, the mixture was shaken in a water bath. Then, KOH solution was introduced for saponification. The saponified mixture was transferred to a separatory funnel, mixed with petroleum ether by shaking, and allowed to stand for phase separation. The organic phase was collected and extracted again using the same procedure. The combined organic phases were washed with water until nearly neutral. After discarding the aqueous phase, the organic extract was evaporated to dryness under reduced pressure and reconstituted in dichloromethane. The resulting solution was filtered through a 0.45 μm membrane prior to instrumental analysis. The analysis was performed on a Thermo U3000 system equipped with a UV detector, a binary gradient pump, and an Acclaim C18 column (4.6 mm × 250 mm, 5 µm). The mobile phase was trichloromethane: acetonitrile: methanol (3: 12: 85, *v*: *v*: *v*), containing 0.4 g/L ascorbic acid. Isocratic elution was applied at a flow rate of 2 mL/min. The column temperature was 35 ± 1 °C, the injection volume was 20 μL, and the detection wavelength was set at 450 nm. β-carotene was identified by comparing its retention time with that of an authentic standard. Quantification was based on an external calibration curve, which was established by plotting the peak area (*y*-axis) against the concentration (*x*-axis) of the standard working solutions. The regression equation was *y* = 0.07 + 1.98*x*, R^2^ = 0.9997, where “*y*” represents the concentration of β-carotene in mg/L, and “*x*” denotes the peak area. The content of β-carotene was expressed in μg/100 g FW. All measurements were conducted in three analytical replicates, and the results are presented as mean ± standard deviation.

### 2.8. Determination of Ascorbic Acid

Ascorbic acid was determined using 2,6-dichlorophenolindophenol (DCPIP) titration and the results were expressed in mg/100 g FW [22]. Briefly, approximately 5 g of homogenized fruit sample was rapidly extracted with oxalic acid solution to prevent ascorbic acid oxidation. The filtrate was titrated against standardized 2,6-dichlorophenolindophenol solution until a faint pink endpoint persisted for 15 s. Blank titration was performed in parallel to correct reagent background. The final ascorbic acid content was calculated according to the titration consumption of DCPIP solution, and all results were expressed as mg/100 g fresh weight (FW). All measurements were conducted in three analytical replicates, and the results are presented as means ± standard deviation.

### 2.9. Determination of Mineral Elements

Mineral elements were determined according to the Chinese national food safety standard GB 5009.268-2025 [23] https://down.foodmate.net/standard/sort/3/162812.html (accessed on 16 October 2024) by microwave digestion–inductively coupled plasma mass spectrometry (ICP-MS). The edible portion was sampled and homogenized thoroughly. Approximately 1 g (±0.01 g) of the sample was weighed into a microwave digestion vessel, and 5 mL of nitric acid was added. The vessel was then placed in a microwave digester for digestion. After digestion was completed, the vessel was transferred to a hot plate to evaporate to near dryness. The residue was dissolved in water and transferred to a 25 mL volumetric flask. Analyses were performed using an ICP-MS instrument (Waltham, MA, USA). The operating parameters were set as follows: RF power was 1550 W; coolant gas flow rate was 14.0 L/min; nebulizer gas flow rate was 1.0 L/min; auxiliary gas flow rate was 0.8 L/min; sample uptake rate was 0.4 mL/min; and sampling depth was 7.8 mm. The calibration standard range was set at 0.05–40 μg/mL for potassium, calcium, sodium, magnesium and aluminum, while the calibration range for zinc, copper, manganese, strontium, boron, titanium, arsenic, lead, mercury, chromium and cadmium was 0.05–40 μg/L. Germanium, rhodium and rhenium were selected as internal standard elements.

The limits of detection (LOD) and limits of quantification (LOQ) for each element were as follows: LOD = 0.3 mg/kg and LOQ = 1 mg/kg for potassium, calcium, sodium, magnesium and iron; LOD = 0.2 mg/kg and LOQ = 0.5 mg/kg for aluminum and zinc; LOD = 0.1 mg/kg and LOQ = 0.3 mg/kg for boron; LOD = 0.02 mg/kg and LOQ = 0.05 mg/kg for copper; LOD = 0.03 mg/kg and LOQ = 0.1 mg/kg for manganese; LOD = 0.05 mg/kg and LOQ = 0.2 mg/kg for strontium; LOD = 0.005 mg/kg and LOQ = 0.02 mg/kg for barium and titanium; LOD = 0.003 mg/kg and LOQ = 0.01 mg/kg for arsenic; LOD = 0.005 mg/kg and LOQ = 0.02 mg/kg for lead; LOD = 0.0003 mg/kg and LOQ = 0.001 mg/kg for mercury; LOD = 0.02 mg/kg and LOQ = 0.05 mg/kg for chromium; LOD = 0.0005 mg/kg and LOQ = 0.002 mg/kg for cadmium. Under repeatability conditions, the absolute difference between two independent determination results should not exceed 20% of their arithmetic mean. All calculated results were expressed in mg/kg. All measurements were conducted in three analytical replicates, and the results are presented as means ± standard deviation.

### 2.10. Determination of Volatile Compounds

Volatile compounds (VOCs) were analyzed using a FlavourSpec GC-IMS system (Gesellschaft für analytische Sensorsysteme mbH, Dortmund, Germany) equipped with a CTC-PAL autosampler (PAL3, CTC Analytics AG, Zwingen, Switzerland). Separation was performed on an MXT-5 capillary column (15 m × 0.53 mm internal diameter., 1.0 µm film thickness; RESTEK, Bellefonte, PA, USA). Briefly, 2 g of homogenized pulp was weighed into a 20 mL headspace vial and sealed. The sample was incubated at 85 °C for 15 min with constant agitation at 500 rpm. The headspace (500 µL) was injected using a syringe. The column temperature was 60 °C and the IMS temperature was 45 °C. Nitrogen was used as the carrier and drift gas. The drift gas flow rate was 150 mL/min. The carrier gas flow rate was programmed as follows: initial flow 2 mL/min (0–2 min), increased to 10 mL/min (2–10 min), and then held at 100 mL/min (10–20 min). For compound identification, retention indices (RIs) were calculated for each signal using a homologous series of n-ketones (C4–C9) analyzed as external references under identical chromatographic conditions. Volatile compounds were identified by comparing the calculated RI and the normalized drift time of each signal with entries in the G.A.S. NIST IMS database; a match was accepted when the RI deviation was less than 10 units and the deviation in normalized drift time was less than 2%. Monomeric and dimeric forms of the same compound (denoted by the suffixes -M and -D, respectively) were assigned according to the library annotations and were retained as separate entries when clearly resolved. Raw data were processed using VOCal software version 0.4.10.

### 2.11. Statistical Analysis

All experiments were performed in triplicate, with composite fruit samples from each orchard defined as the experimental unit. Results were expressed as mean ± standard deviation. One-way analysis of variance (ANOVA) was performed using SPSS 26, followed by Duncan’s multiple range test. Differences at *p* < 0.05 were considered statistically significant. Pearson correlation analysis was used to evaluate relationships among quality parameters and between quality traits and environmental factors. Principal component analysis (PCA) was performed using Origin 2021. Orthogonal partial least squares discriminant analysis (OPLS-DA) was carried out using SIMCA 14.1 to explore sample similarities and differences. All variables were preprocessed by mean centering and unit variance (UV) scaling prior to modeling. R^2^X, R^2^Y and Q^2^ were used to assess the fitting and predictive performance of the OPLS-DA model. A permutation test with 200 iterations was conducted to exclude the risk of model overfitting. The number of components was determined by cross-validation and model interpretability. Radar charts and column charts were plotted using Origin 2021.

## 3. Results

### 3.1. Physicochemical Properties

#### 3.1.1. Phenotypic Characteristics and Sensory Evaluation

**Figure 3:**
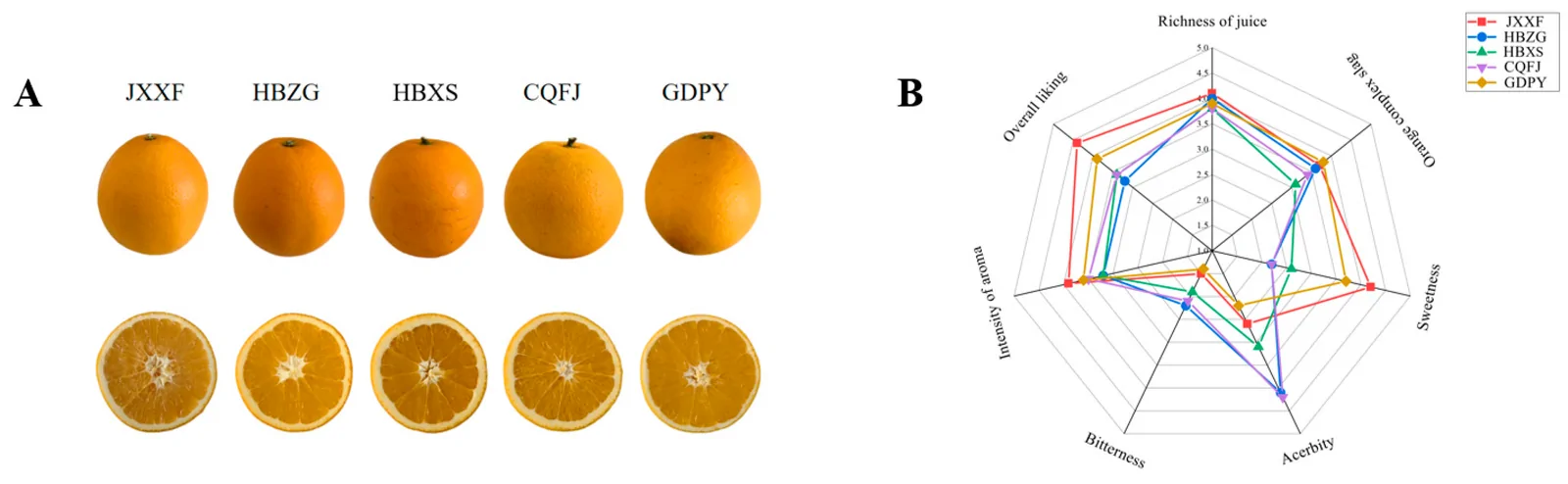
Sensory evaluation of Newhall navel oranges in different producing regions. (**A**) Fruit appearance and profile map. (**B**) Sensory characteristics radar chart.

Sensory evaluation plays an essential role in assessing fruit quality, as it directly mirrors consumers’ real-world perceptual experiences [24]. Newhall navel oranges from the five producing regions exhibited similar fruit sizes and varying shapes (Figure 3A). Fruits from JXXF, HBZG, and GDPY displayed a distinctly oblong shape, whereas those from CQFJ and HBXS were more spherical. Additionally, fruits from JXXF had a noticeably smoother peel surface.

Fruit color is a key quality trait influencing both consumer preference and juice processing [25]. Newhall navel oranges from HBZG, GDPY, and JXXF exhibited higher peel L* values, while those from HBZG had the highest peel b* value, indicating a more yellowish appearance, which was consistent with the sensory evaluation results (Table 2). Oranges from CQFJ showed the highest pulp L*, a*, and b* values, indicating the greatest brightness and yellowness in pulp color. Fruit coloration is influenced by the combined effects of pigments such as chlorophyll and carotenoids. Among these, the composition and content of carotenoids are primarily responsible for yellow and orange coloration in fruits [26].

The sensory evaluation radar chart revealed the following overall preference ranking: JXXF > GDPY > CQFJ = HBXS > HBZG (Figure 3B). Newhall navel oranges from JXXF were the most preferred by consumers, with a comprehensive score of 3.8, characterized by moderate acidity, low bitterness, minimal residue, and a strong aroma. Those from GDPY received the highest score (3.8) for ease of segment separation (residue minimization), whereas the low overall preference for HBZG oranges may be attributed to pronounced acidity and bitterness (scores of 4.1 and 2.2, respectively), resulting in a less desirable taste profile. These results suggest that the balance between sweetness and acidity, along with the level of residue, was a key factor influencing consumer preference in Newhall navel oranges. HBXS oranges had the highest RTT (26.8), followed by JXXF (22.2), while HBZG had the lowest (12.55) (Table 2).

#### 3.1.2. Peel Thickness, Edible Rate and Juice Yield

Peel thickness of Newhall navel oranges from the five producing regions ranged from 0.38 cm to 0.56 cm (Table 2). The GDPY fruits had the thinnest peel (0.38 ± 0.05 cm), whereas those from CQFJ had the thickest peel (0.56 ± 0.03 cm). The edible rate ranged from 71.10% to 83.67%, with the highest value observed in HBZG fruits (83.67 ± 2.49%) and the lowest in JXXF fruits (71.10 ± 3.75%). Notably, the correlation between peel thickness and edible rate was inconsistent, suggesting that this relationship may be mediated by other factors such as fruit weight, moisture content, and seed number [27]. The juice yield ranged from 39.68% to 44.70%, with relatively higher yields observed in Newhall navel oranges from HBXS, CQFJ, and GDPY, whereas HBZG and JXXF fruits had slightly lower juice yields.

#### 3.1.3. TSS, SS, TA and OA Content

**Figure 4:**
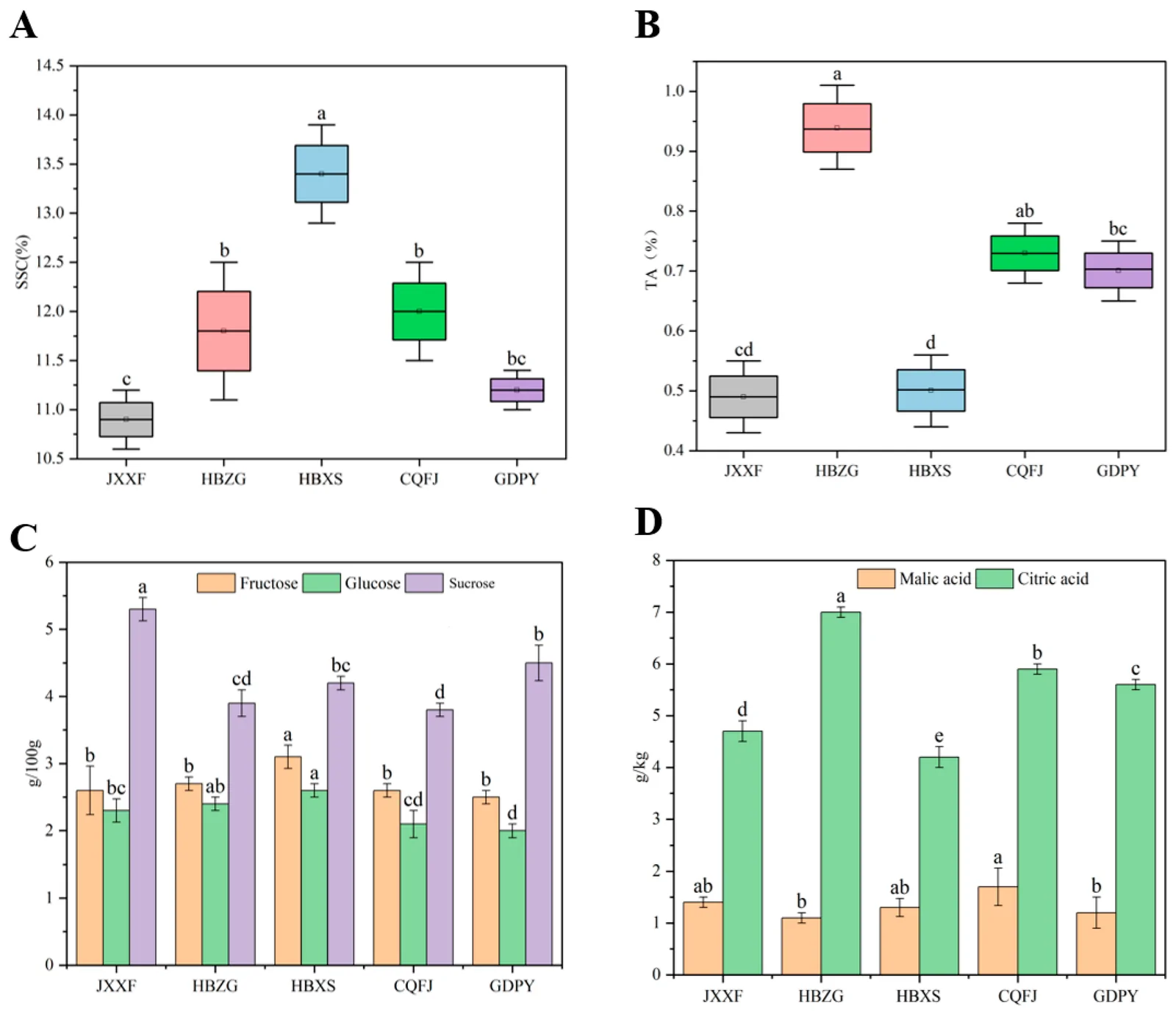
Sugar and acid components in Newhall navel oranges in different producing regions. (**A**) SSC: Soluble solids content. (**B**) TA: Titrable acid. (**C**) SS: Soluble sugar. (**D**) OA: Organic acids. Note: Different lowercase letters indicate significant differences among samples (*p* < 0.05).

Sugar and acid content are key factors influencing the sensory quality of fruits and directly determine consumer acceptability [22]. The SSC of Newhall navel oranges from the five major producing regions in China all exceeded 10%, with values ranging from 10.9% to 13.4% (Figure 4A). The highest SSC was observed in HBXS (13.4%), followed by CQFJ (12.0%) and HBZG (11.8%), while GDPY (11.2%) and JXXF (10.9%) were comparatively lower. Significant differences were observed in TA content among regions (Figure 4B), with values ranging from 0.49% to 0.94%. The highest TA was found in HBZG (0.94%), followed by CQFJ (0.73%) and GDPY (0.70%), whereas HBXS (0.50%) and JXXF (0.49%) had relatively lower TA levels, which were largely consistent with the sensory evaluation results. Fructose, glucose, and sucrose are the major sugars in navel oranges [28]. Sucrose was the most abundant soluble sugar, with content exceeding 3.8 g/100 g in all regions (Figure 4C). The ranking of sucrose content was JXXF > GDPY > HBXS > CQFJ, with JXXF having the highest value (5.3 g/100 g) and CQFJ the lowest (3.8 g/100 g). The other three regions exhibited intermediate sucrose levels. The high sucrose content contributed to a sweeter taste and milder bitterness, aligning with the sensory evaluation outcomes. Glucose and fructose content was highest in the HBXS samples, reaching 2.6 g/100 g and 3.1 g/100 g, respectively, with no significant differences among the other four regions. Citric acid content showed the most significant variation (*p* < 0.05) among the five regions, with the highest level observed in HBZG (7.0 g/kg), followed by CQFJ (5.9 g/kg) and GDPY (5.6 g/kg) (Figure 4D). The lowest values were recorded in JXXF (4.7 g/kg) and HBXS (4.2 g/kg). In contrast, malic acid content exhibited small but significant differences, with values ranging from 1.1 to 1.7 g/kg. These findings are consistent with previous reports [29]. In summary, Newhall navel oranges can be characterized as sucrose and citric-acid-accumulating fruits. Fruits with low bitterness and high sweetness are more suitable for fresh consumption, whereas those with higher acidity may be better suited for processing.

### 3.2. Nutritional Properties

#### 3.2.1. Hesperidin, Ascorbic Acid, and β-Carotene Content

**Figure 5:**
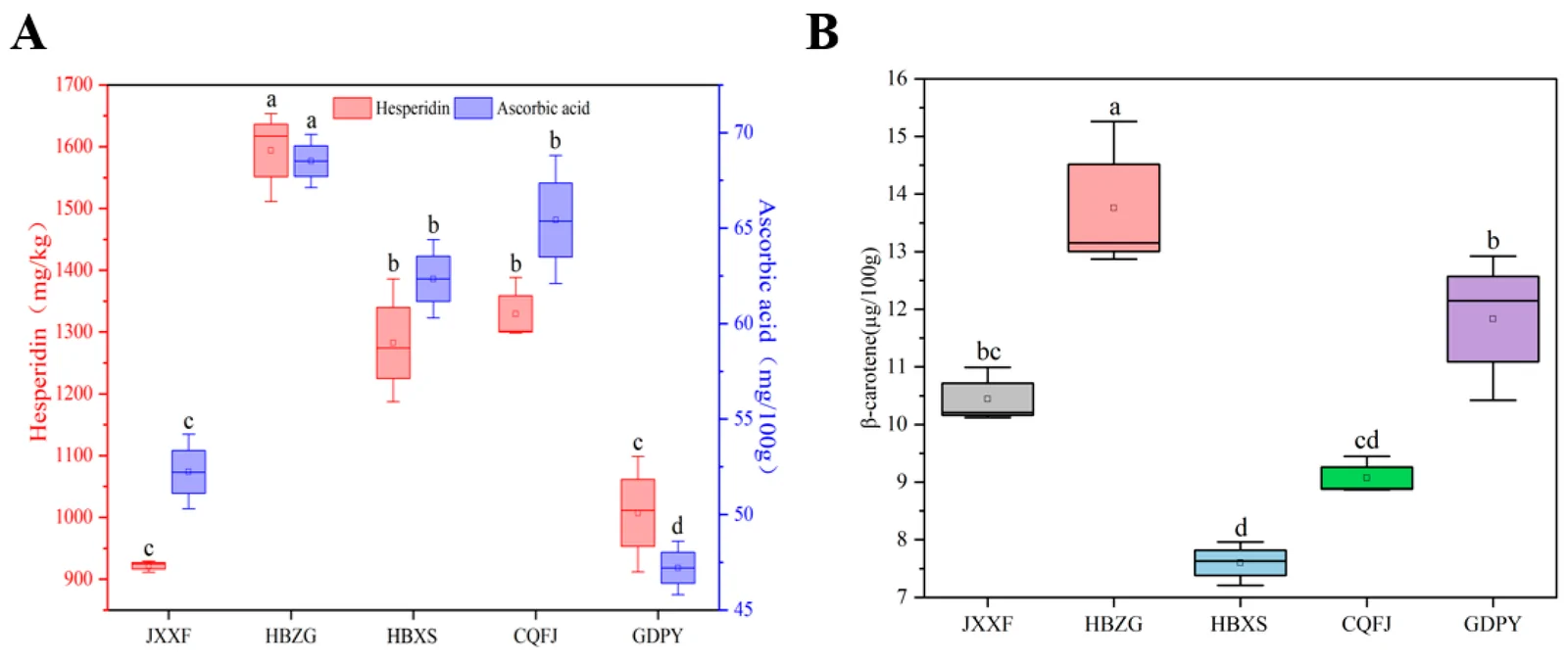
Functional components in Newhall navel oranges in different producing regions. (**A**) Hesperidin and ascorbic acid. (**B**) β-carotene. Note: Different lowercase letters indicate significant differences among samples (*p* < 0.05).

Hesperidin and ascorbic acid are key antioxidant constituents in Newhall navel oranges, underpinning the fruit’s antioxidant capacity and associated health-promoting properties [30]. HBZG samples had the highest levels of both hesperidin and ascorbic acid, at 1593.81 mg/kg and 68.5 mg/100 g, respectively (Figure 5A). CQFJ and HBXS exhibited intermediate content, while the lowest values were found in GDPY and JXXF. Notably, although the ascorbic acid content in JXXF (52.2 mg/100 g FW) was higher than that in GDPY (47.2 mg/100 g FW), the hesperidin content in JXXF was the lowest among all regions, at only 921.45 mg/kg. Hesperidin is synthesized primarily via the phenylpropanoid pathway, with phenylalanine and malonyl-CoA as precursors, under the catalysis of phenylalanine ammonia-lyase (PAL) and chalcone synthase (CHS). Conversely, ascorbic acid is mainly biosynthesized via the L-galactose pathway, starting from D-glucose and involving key enzymes such as L-galactono-1,4-lactone dehydrogenase (GLDH) [31]. β-carotene is a stable orange-yellow natural pigment widely present in vegetables and orange or yellow-colored fruits. This may explain the more yellowish peel color observed in the sensory evaluation of fruits from HBZG [32]. The β-carotene content in pulp was highest in HBZG (13.76 µg/100 g), followed by GDPY (11.83 µg/100 g) and JXXF (10.44 µg/100 g), while HBXS (7.60 µg/100 g) and CQFJ (9.07 µg/100 g) contained relatively lower levels (Figure 5B). β-carotene is synthesized through the carotenoid pathway, with catalysis by phytoene synthase (PSY), and plays important roles in preventing chronic diseases, enhancing immunity, and reducing the risk of cardiovascular diseases [33]. Therefore, Newhall navel oranges from HBZG are rich in hesperidin, ascorbic acid, and β-carotene. Fruits with high bioactive compound contents may be more advantageous for the development of functional orange juice products [34].

#### 3.2.2. Mineral Elements Content

Newhall navel oranges are rich in mineral elements, which play vital roles in human health. Previous studies have primarily focused on nutrients such as sugars, acids, and polyphenols in Newhall navel oranges, whereas relatively few studies have investigated mineral elements. A total of 12 mineral elements and five heavy metals were determined (Table 3). Potassium (K), calcium (Ca), and magnesium (Mg) were the most abundant minerals. Among them, the highest K content was found in samples from CQFJ (1239.28 ± 8.28 mg/kg), followed by HBXS (1112.15 ± 9.15 mg/kg) and GDPY (1075.13 ± 10.13 mg/kg), while HBZG (994.63 ± 13.37 mg/kg) and JXXF (989.17 ± 11.17 mg/kg) exhibited relatively lower values. The highest contents of both Ca and Mg were detected in the HBXS samples, at 417.73 ± 9.27 mg/kg and 108.66 ± 2.66 mg/kg, respectively. In contrast, JXXF exhibited lower levels of K and Mg. Regarding trace elements, the HBXS samples exhibited notably higher levels of Fe (5.06 ± 0.17 mg/kg), Zn (0.87 ± 0.02 mg/kg), Mn (1.40 ± 0.10 mg/kg), Sr (1.23 ± 0.05 mg/kg), and B (0.96 ± 0.01 mg/kg). Furthermore, the concentrations of heavy-metal elements including arsenic, lead, mercury, and chromium in samples from all five producing regions were below their respective LOD. The mineral content of fruits and vegetables is influenced by factors such as cultivar, soil and climate conditions, soil quality, harvest maturity, and fertilization practices [35]. The HBXS region is characterized by extensive karst topography, with water sources primarily derived from groundwater or that flow through limestone strata, resulting in elevated concentrations of calcium and magnesium ions. In contrast, the sampling site of JXXF lies within hilly and mountainous terrain. Based on regional geological background information, local soils are reported to have relatively low fertility and organic matter content, which may limit nutrient uptake by fruit trees. In addition, the dominant red soils in this area have been documented to be slightly acidic (pH ≈ 4.8), a property that could reduce the bioavailability of elements including calcium (Ca), phosphorus (P), and magnesium (Mg). Furthermore, improper fertilization practices may lead to antagonistic effects that further inhibit mineral uptake [14]. Studies have shown that macronutrients such as K and Ca, along with micronutrients like Zn and B, are essential for maintaining fruit quality. An imbalance in these nutrients can compromise the structural integrity of plant cells, weaken biochemical defense systems, and disrupt normal physiological processes, thereby increasing susceptibility to diseases [36].

### 3.3. Quality Correlation Analysis and PCA

**Figure 6:**
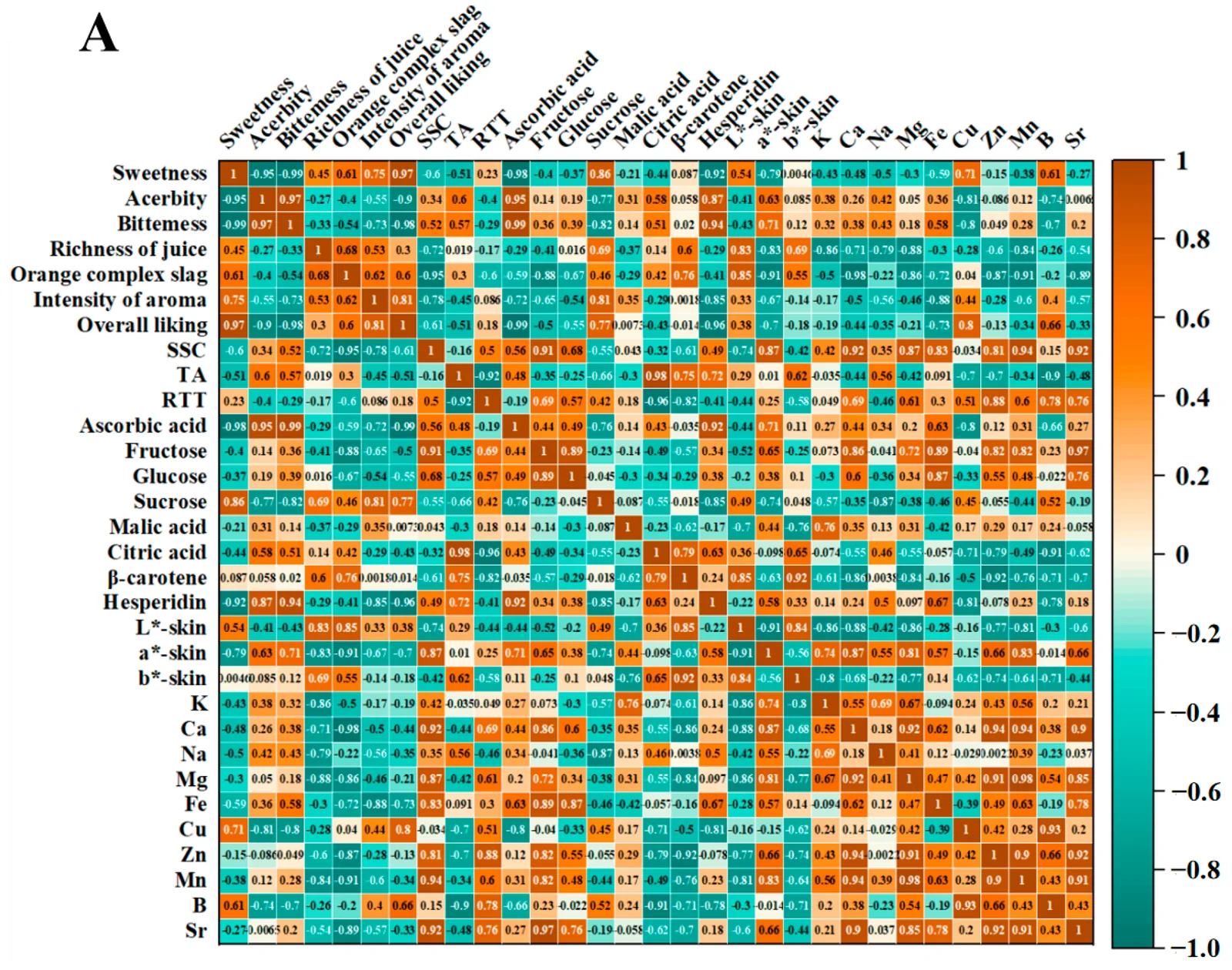

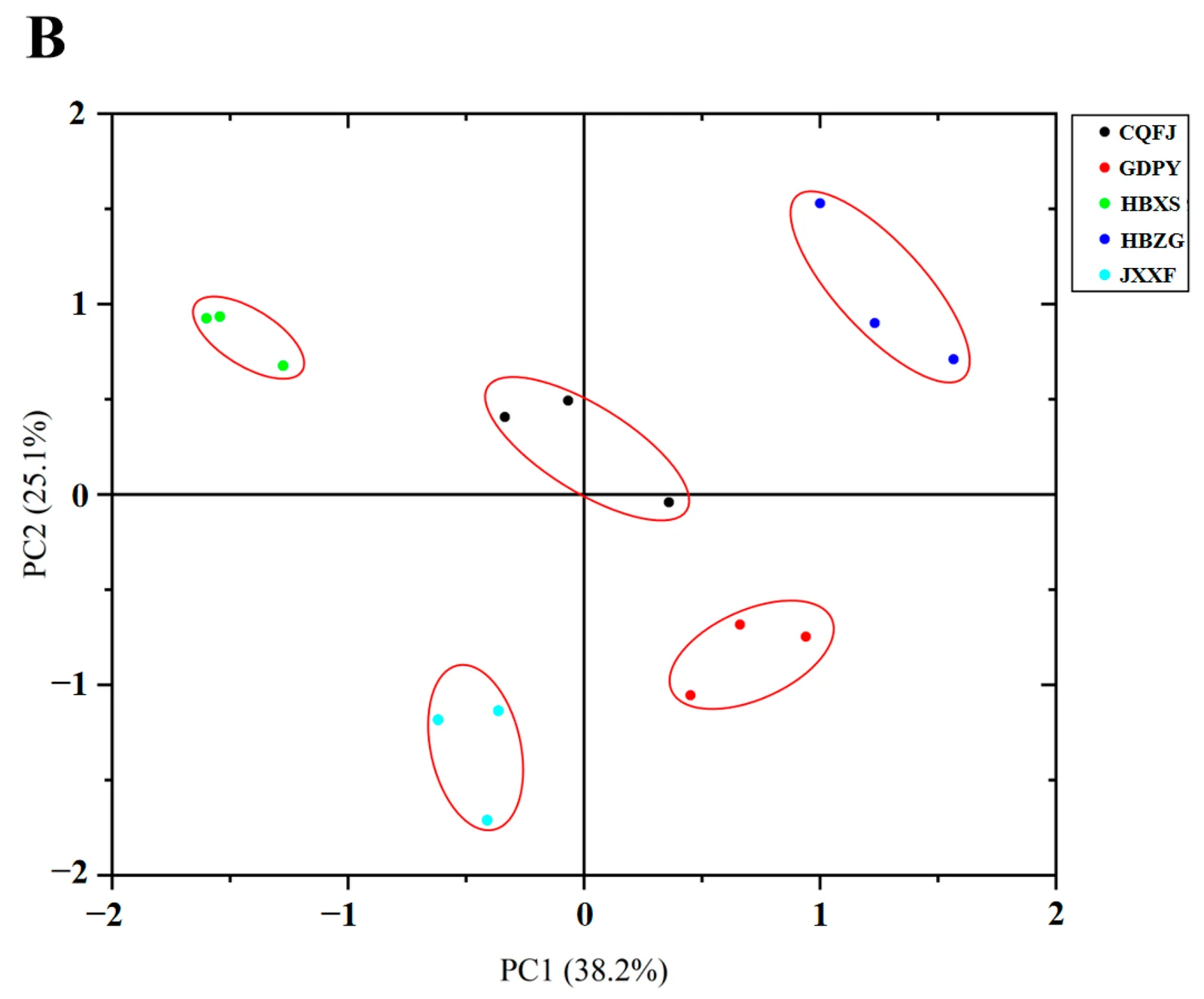
Analysis of correlations between sensory and nutritional indicators, and principal component analysis of Newhall navel oranges. (**A**) Correlation heat map. (**B**) Principal component analysis. The red circles are drawn only for visual discrimination of sample groups and have no statistical significance.

The correlation analysis between nutritional components and sensory evaluation is presented in Figure 6A. Sweetness exhibited a strong positive correlation with sucrose (r = 0.86), while showing significant negative correlations with citric acid and titratable acidity, reflecting the crucial regulatory role of sugar–acid balance in overall flavor perception. Furthermore, sucrose not only correlated positively with sweetness but also displayed negative correlations with acerbity and bitterness, as well as positive correlations with aroma intensity and overall liking, indicating its potential as a key indicator for evaluating Newhall navel oranges’ flavor quality. Both ascorbic acid and hesperidin were negatively correlated with aroma intensity and overall liking, but positively correlated with acerbity and bitterness, suggesting their significant influence on the flavor profile of Newhall navel oranges. Additionally, their positive correlation with the a* value (red–green chromaticity) further implies that these two components may serve as key functional factors linking appearance quality to flavor attributes. Soluble solids constitute an important indicator for characterizing fruit flavor. RTT is closely correlated with sensory perception and flavor quality. RTT shows positive correlations with fructose and soluble solids, a positive correlation with sweetness (r = 0.23), and a negative correlation with acidity (r = −0.4). PCA is a widely used tool for exploring similarities and differences among samples by reducing data dimensionality and integrating variables [37]. The first two principal components, PC1 and PC2, accounted for 38.2% and 25.1% of the total variance, respectively, collectively representing a substantial portion of the dataset variability (Figure 6B). PC1 was primarily influenced by titratable acidity, citric acid, β-carotene, and edible rate, whereas PC2 was mainly associated with soluble solids, fructose, sucrose, glucose, and hesperidin. Samples from HBZG, JXXF, HBXS, and GDPY were clearly separated along PC1 and PC2, while those from CQFJ clustered in the middle, indicating that sugar and acid components effectively discriminated samples from these regions.

### 3.4. Volatile Aroma Components

**Figure 7:**
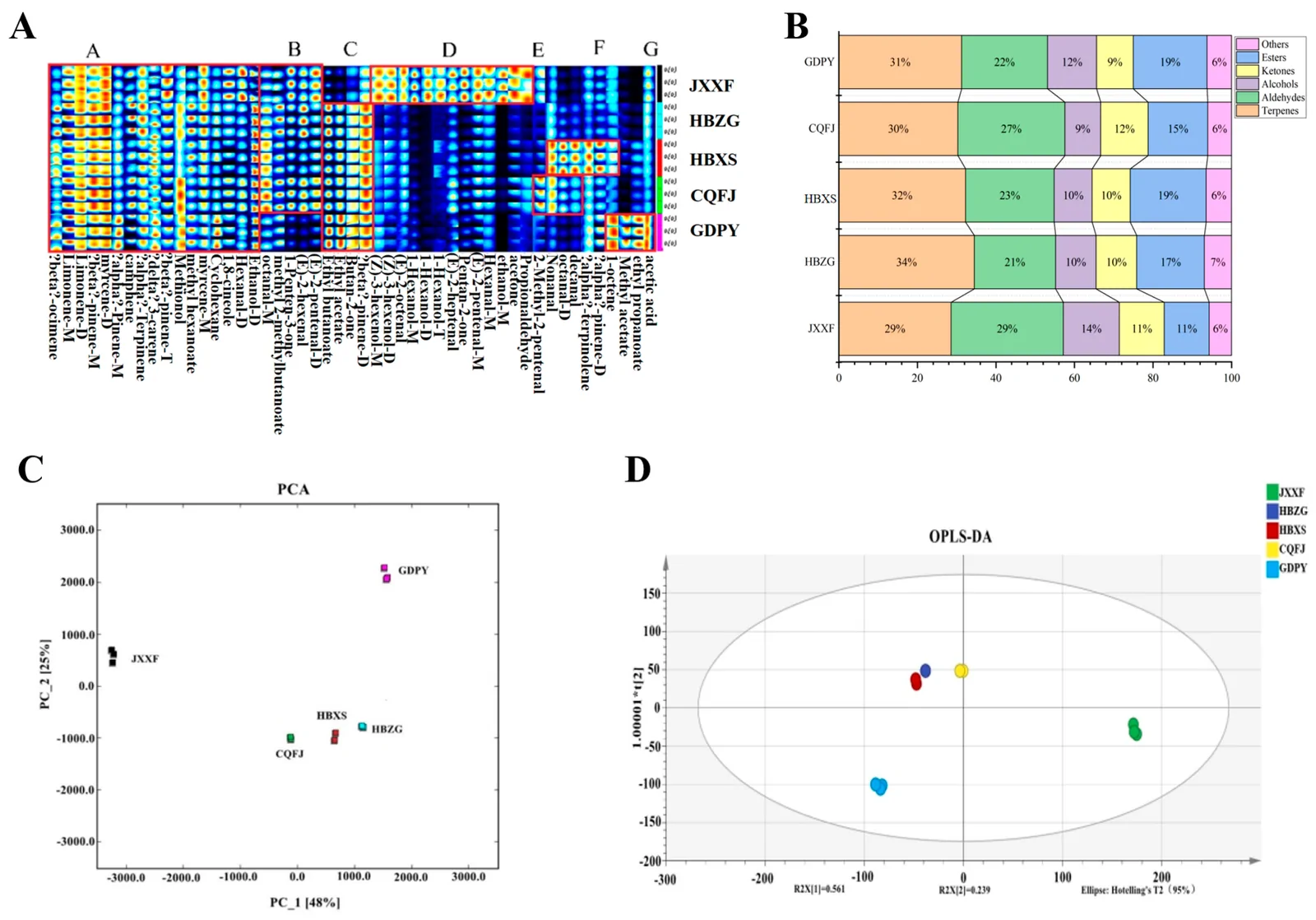

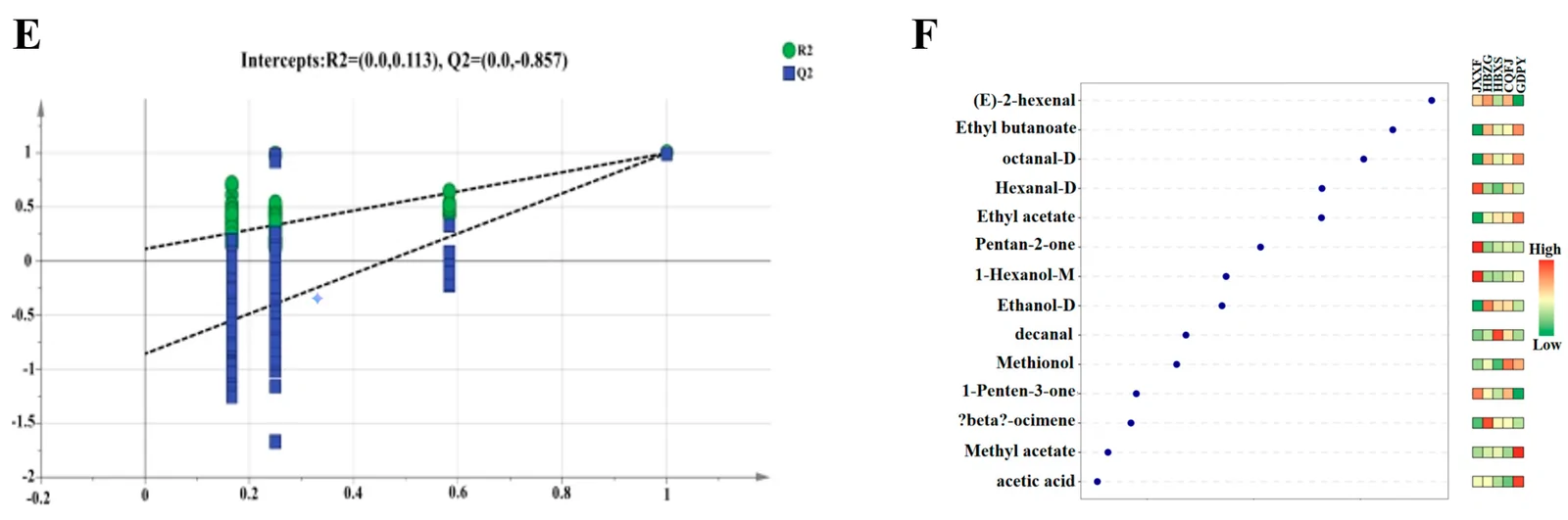
GC-IMS fingerprinting patterns and data analysis. (**A**) Fingerprints of volatile compounds in Newhall navel oranges with different regions. (**B**) Percentage of aroma substances in Newhall navel orange in different producing regions. (**C**) Principal component analysis diagram of aroma scores plot. (**D**) OPLS-DA. (**E**) OPLS-DA 200 times permutation testing. The dashed lines represent regression lines of permuted R^2^ (green) and Q^2^ (blue). (**F**) VIP > 1 aroma compound substances.

A total of 49 volatile compounds (including monomers and dimers) were detected in Newhall navel orange samples from five producing regions using HS-GC-IMS (Figure S1 and Table S3 in Supplementary Materials). These included 15 terpenes, 13 aldehydes, nine alcohols, six esters, four ketones, and two other compounds. A fingerprint profile of the volatile compounds in different regions was generated using the Gallery Plot plugin (Figure 7A), with the *x*-axis representing compound names, the *y*-axis indicating producing regions, and color intensity reflecting the relative abundance of each volatile compound [38]. Newhall navel oranges from JXXF contained a relatively higher diversity and relative abundance of volatile compounds, with elevated levels of (E)-2-octenal, 2-pentanone, 2-methyl-2-pentenal, leaf alcohol (3-hexen-1-ol), hexanal, hexanol, and (E)-2-heptenal. Among these, (E)-2-octenal, hexanal, leaf alcohol, and hexanol correlate with “green grassy” and “sweet aromatic” notes (Figure 7A). The combination of these aromas gave JXXF oranges a stronger “green–sweet” fragrance [39]. It was worth noting that myrcene showed the highest relative abundance in samples from JXXF. Accumulation of myrcene may enhance resistance against pathogens, reducing susceptibility to infection or infestation [40]. However, the relative abundance of some compounds such as ethyl butyrate, ethyl acetate, and β-pinene were lower compared to those in other producing regions. Newhall navel oranges from the HBZG, HBXS, and CQFJ regions showed high similarity in volatile composition. In addition, those from HBXS contained higher levels of nonanal, decanal, octanal, terpinolene, and α-pinene, correlating with a prominent citrus and fatty aroma. CQFJ samples were characterized by a noticeable fruity scent, mainly due to greater relative abundance of 2-methyl-2-pentenal and nonanal. In contrast, GDPY Newhall navel oranges exhibited an overall citrus-like and intense fruity aroma, with methyl acetate and ethyl propionate being unique to this region. Terpenes were the most abundant class, accounting for 29–34% of the total relative abundance and serving as the major contributors to the aroma profile of Newhall navel oranges (Figure 7B). Aldehydes (21–29%) and esters (11–19%) were the next most predominant categories. Similar results were also found in other oranges [3]. To further explore regional differences, PCA was performed in this work. PC1 and PC2 accounted for 60.19% and 23.09% of the total variance, respectively, cumulatively explaining 83% of the variability, indicating that the two components effectively captured the dominant features of the samples (Figure 7C). The JXXF samples were most separated from others based on the analysis of PC1, while GDPY showed the greatest separation along PC2, suggesting that these two regions possess distinctive aroma profiles compared to the others. In contrast, samples from HBZG, HBXS, and CQFJ clustered closely on the PCA plot with small inter-group distances along PC2, indicating minimal differences in flavor profiles among these three regions. In the OPLS-DA model (Figure 7D), R^2^Y and Q^2^ are commonly used to evaluate model stability and data reliability. Values closer to 1 indicate a more robust model. The obtained values of R^2^Y = 0.988 and Q^2^ = 0.978 demonstrated that the model effectively explained the variability among samples and highlighted clear separation between the five producing regions. To prevent overfitting, a permutation test with 200 iterations was performed (Figure 7E). The resulting Q^2^ < 0 confirmed that no overfitting occurred [41]. To further identify discriminant markers for the five regions, variable importance in projection (VIP) scores from the OPLS-DA model were used, and compounds with VIP > 1 were selected for analysis. Since VIP values > 1.5 were generally considered more significant, (E)-2-hexenal (imparting a strong, fresh fruity and green leafy aroma), ethyl butanoate (fruity scent), octanal-D (sharp green waxy note), hexanal-D (green, grassy, and apple-like aroma), ethyl acetate (fruity fragrance), and pentan-2-one (woody note), which all exhibited VIP values > 1.5 and distinct regional differences in peak volume, were identified as key aroma markers for geographical discrimination (Figure 7F). The formation of these aroma markers was closely associated with metabolic pathways such as fatty acid oxidation and ester synthesis in Newhall navel oranges. Aldehydes and ketones responsible for green and leafy notes primarily originate from the fatty acid oxidation pathway. This process began with unsaturated fatty acids such as linoleic and linolenic acid, which were oxidatively degraded into short-chain volatile aldehydes under the catalysis of lipoxygenase (LOX) and hydroperoxide lyase (HPL). Specifically, hexanal-D and (E)-2-hexenal were direct products of this pathway and served as core contributors to the “green” aroma. In contrast, octanal-D was mainly generated via α-oxidation of saturated fatty acids such as palmitic acid, contributing a distinctive waxy and citrus-peel note [42]. On the other hand, fruity-smelling esters such as ethyl butanoate and ethyl acetate were largely synthesized through the catalytic activity of alcohol acyltransferase (AAT), which facilitated the combination of acyl-CoA and ethanol to form esters [43].

### 3.5. Analysis of the Correlation Between Newhall Navel Orange Quality and Environmental Factors

**Figure 8:**
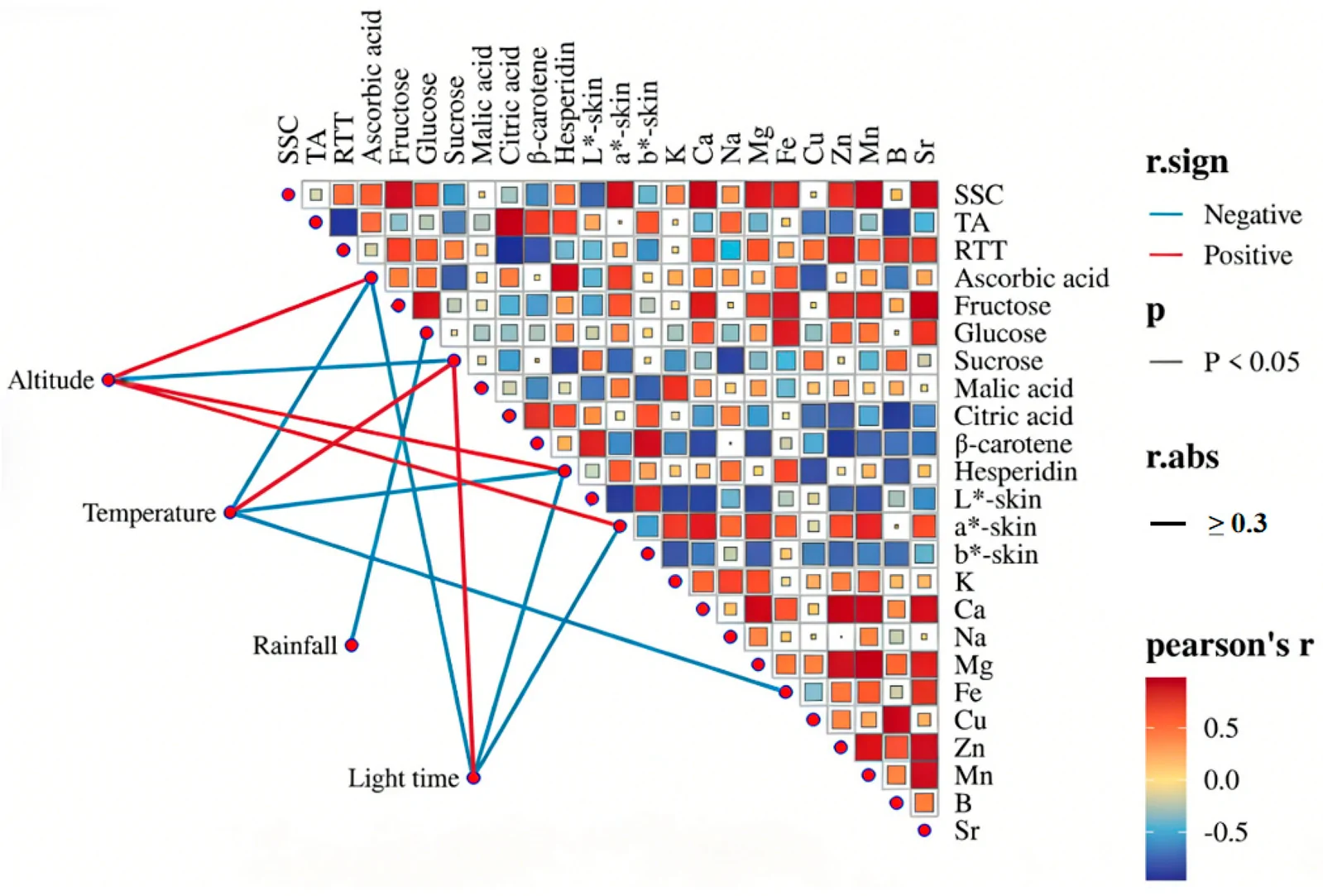
An analysis of the correlation between environmental factors and the quality of Newhall navel oranges.

The quality of Newhall navel oranges is influenced by soil, climate, topography, and other environmental factors [6,7]. To establish relationships between environmental variables and fruit quality parameters, correlation analysis was conducted on altitude, average temperature, rainfall, and sunlight hours (from bud burst to harvest). The red solid line represented a significant positive correlation, while the blue solid line represented a significant negative correlation (Figure 8). For these quality parameters, the a* value was positively correlated with altitude (*p* < 0.05, r > 0.3) and negatively correlated with sunlight hours (*p* < 0.05, r > 0.3). The contents of ascorbic acid and hesperidin were positively correlated with altitude, but negatively correlated with temperature and sunlight hours (*p* < 0.05, r > 0.3). Regions such as CQFJ and HBZG, characterized by higher altitudes and lower temperatures (Figure 2), exhibited higher ascorbic acid content. In contrast, the high temperature and humidity during summer in GDPY may accelerate the degradation of ascorbic acid in the late stages of fruit development or before harvest [44]. Higher altitudes are associated with stronger ultraviolet radiation and lower temperatures, leading to increased reactive oxygen species (ROS). This oxidative stress acts as a signal inducing antioxidant metabolism, upregulating key genes involved in ascorbic acid synthesis and enhancing the phenylpropanoid pathway, thereby promoting the production of more antioxidants [45]. Rokaya [46] reported that citrus fruits grown at 1500 m altitude contained significantly higher ascorbic acid than those at 700 m, which was consistent with the findings of this study. Glucose content was negatively correlated with rainfall, but sucrose was positively correlated with temperature and light time (*p* < 0.05, r > 0.3). The sufficient sunlight and large diurnal temperature variation in JXXF facilitated sugar accumulation. During rainy periods, photosynthetic activity was diminished, which limited sugar accumulation. Additionally, the activity of citrate degrading enzymes decreased significantly under conditions of low temperature, high humidity, and hypoxia, hindering the entry of synthesized citric acid into the TCA cycle and resulting in a fruit quality profile characterized by “high acid and low sugar” [47]. In HBZG, heavy precipitation during the fruit expansion stage (July and August) was unfavorable for sugar accumulation and may have promoted organic acid accumulation.

It should be noted that only one representative orchard was sampled within a single growing season for each region in this study. Therefore, the observed associations reflect the conditions of the selected orchards. These correlation results are exploratory rather than definitive conclusions and future work with more orchards, multiple seasons, and independent biological replicates will be needed to further verify these environment–quality relationships.

## 4. Conclusions

This study analyzed the characteristic differences of Newhall navel oranges from five major producing regions in China. The results indicated that Newhall navel oranges are a sucrose- and citric-acid-accumulating fruit. The oranges from JXXF were characterized by low acidity, high sucrose content, and abundant volatile aroma compounds, making them highly preferred by consumers. In contrast, those from HBZG exhibited high acidity and elevated levels of functional components such as ascorbic acid, hesperidin, and β-carotene, suggesting greater suitability for juice processing. No significant difference was observed between HBXS and CQFJ, while GDPY showed moderate overlap with these regions. Using an OPLS-DA model, VIP analysis identified six volatile markers with VIP > 1.5, namely (E)-2-hexenal, ethyl butanoate, octanal-D, hexanal-D, ethyl acetate, and pentan-2-one, which could effectively discriminate the geographical origins of the samples. The quality variations of the Newhall navel oranges exhibited close associations with climatic conditions. Higher elevation may be correlated with differences in fruit coloration and the accumulation of ascorbic acid and hesperidin, while greater precipitation may be associated with lower sugar accumulation. The study provides an important reference for identifying producing region differences and guiding consumer choices, as well as insights into optimized cultivation strategies for Newhall navel oranges under climate change.

## Figures and Tables

**Table 1:** Specific geographical location information of the Newhall navel orange producing regions.

Regions	Longitude	Latitude	Average Altitude (m)
JXXF	114°55′6.0″ E	25°23′19.9″ N	319
HBZG	110°58′21.1″ E	30°49′39.3″ N	616
HBXS	111°16′51.8″ E	30°41′40.9″ N	592
CQFJ	109°24′20.2″ E	31°1′23.8″ N	654
GDPY	115°53′12.0″ E	24°34′11.4″ N	362

**Table 2:** A comparison of the peel thickness, edible rate, juice yield, RTT and the color of the skin and flesh of Newhall navel oranges in different producing regions.

Regions	Thickness/cm	Edible Rate/%	Juice Yield/%	RTT	Color Indicators of Skin			Color Indicators of Pulp		
					L*	a*	b*	L*	a*	b*
JXXF	0.50 ± 0.03 b	71.10 ± 2.74 d	41.89 ± 3.75 b	22.24	66.3 ± 0.40 a	19.6 ± 0.40 d	62.9 ± 0.80 b	35.1 ± 0.40 c	1.9 ± 0.10 c	11.8 ± 0.40 e
HBZG	0.45 ± 0.06 c	83.67 ± 2.49 a	39.68 ± 7.53 b	12.55	67.0 ± 0.40 a	27.8 ± 0.70 b	70.3 ± 1.10 a	38.4 ± 1.10 ab	1.5 ± 0.10 d	18.9 ± 0.60 a
HBXS	0.55 ± 0.02 a	73.12 ± 4.74 d	44.70 ± 4.81 a	26.80	58.6 ± 0.50 b	38.4 ± 1.00 a	58.1 ± 1.30 c	37.1 ± 1.10 b	3.2 ± 0.00 b	16.9 ± 0.30 b
CQFJ	0.56 ± 0.05 a	75.60 ± 3.71 c	42.14 ± 3.27 a	16.44	57.7 ± 0.80 b	37.7 ± 0.80 a	56.9 ± 0.90 c	38.8 ± 0.70 a	3.5 ± 0.10 a	19.1 ± 0.30 a
GDPY	0.38 ± 0.05 d	79.74 ± 5.47 b	42.27 ± 2.32 a	16.00	66.5 ± 0.60 a	21.5 ± 0.60 c	62.6 ± 0.50 b	38.4 ± 0.10 ab	1.2 ± 0.10 e	13.7 ± 0.40 c

Note: Data are expressed as means ± standard deviation (SD). Different lowercase letters in the same column indicate significant differences among samples (*p* < 0.05).

**Table 3:** Types and contents of minerals in Newhall navel oranges in different producing regions (mg/kg).

Regions	K	Ca	Na	Mg	Al	Fe	Zn	Cu	Mn	Sr	B	Ti
JXXF	989.17 ± 11.17 e	172.62 ± 3.62 c	1.48 ± 0.09 e	74.42 ± 3.22 d	0.13 ± 0.01 c	1.17 ± 0.10 d	0.54 ± 0.01 c	0.35 ± 0.01 c	0.44 ± 0.02 e	0.21 ± 0.02 d	0.85 ± 0.00 b	0.12 ± 0.02 c
HBZG	994.63 ± 13.37 d	165.55 ± 6.55 d	4.04 ± 0.06 c	74.24 ± 1.64 d	0.37 ± 0.01 b	4.04 ± 0.08 b	0.40 ± 0.01 e	0.23 ± 0.02 d	0.57 ± 0.01 d	0.26 ± 0.01 c	0.30 ± 0.02 d	0.11 ± 0.02 c
HBXS	1112.15 ± 9.15 b	417.73 ± 9.27 a	3.82 ± 0.31 d	108.66 ± 2.66 a	0.00 ± 0.00 d	5.06 ± 0.17 a	0.87 ± 0.02 a	0.37 ± 0.01 b	1.40 ± 0.10 a	1.23 ± 0.05 a	0.96 ± 0.01 a	0.28 ± 0.02 a
CQFJ	1239.28 ± 8.28 a	287.26 ± 13.26 b	5.12 ± 0.14 a	93.62 ± 1.92 b	4.75 ± 0.16 a	1.57 ± 0.09 c	0.60 ± 0.02 b	0.32 ± 0.06 c	0.89 ± 0.04 b	0.28 ± 0.01 b	0.67 ± 0.05 c	0.20 ± 0.01 b
GDPY	1075.13 ± 10.13 c	137.45 ± 3.55 e	4.61 ± 0.24 b	84.77 ± 1.97 c	0.00 ± 0.00 d	1.17 ± 0.11 d	0.49 ± 0.01 d	0.41 ± 0.03 a	0.69 ± 0.00 c	0.19 ± 0.01 e	0.89 ± 0.05 b	0.09 ± 0.02 d

Note: K: potassium; Ca: calcium; Na: sodium; Mg: magnesium; Al: aluminum; Fe: iron; Zn: zinc; Cu: copper; Mn: manganese; Sr: strontium; B: boron; Ti: titanium; As: arsenic; Pb: lead; Hg: mercury; Cr: chromium; Cd: cadmium. Data are expressed as means ± standard deviation (SD). Different lowercase letters in the same column indicate significant differences among samples (*p* < 0.05).

## Data Availability

The original contributions presented in this study are included in the article/Supplementary Materials. Further inquiries can be directed to the corresponding authors.

## Supplementary Materials

The following supporting information can be downloaded at https://www.mdpi.com/article/10.3390/foods15213792/s1, Figure S1: GC-IMS analysis of five different Newhall navel oranges producing regions; Table S1: Reference standards for the evaluation of Newhall navel oranges sensory attributes by trained panels; Table S2: Sensory evaluation scores of Newhall navel oranges from different producing regions; Table S3: Newhall navel oranges aroma substances in different producing regions.
