# Supplementary material for: Geographic Regions and Environmental Factors Influence the Nutritional and Flavor Quality of Newhall Navel Oranges
Source: Foods. 2026 Oct 23;15(21):3792. doi: 10.3390/foods15213792 (PMC13762235; doi:10.3390/foods15213792)
Supplement: Supplementary file 1 [file foods-15-03792-s001.zip › Supplementary Material 1.pdf]

SUPPLEMENTARY MATERIAL

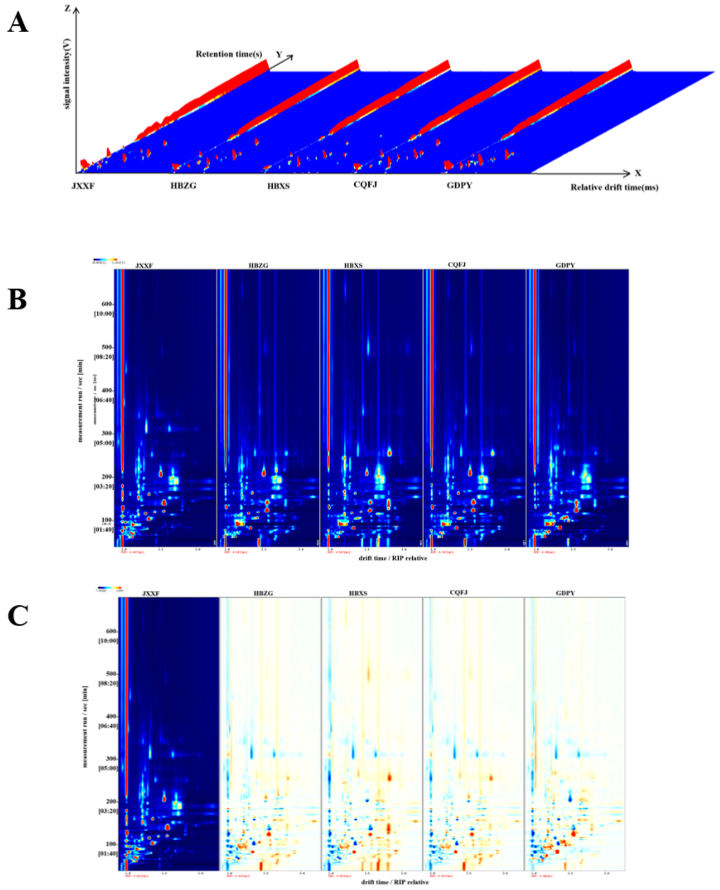

Figure.S1. GC-IMS analysis of five different Newhall navel oranges producing regions. (A) 3D-topographic. (B) 2D-topographic plots. (C) The difference comparison topographic plot.
